# Supplementary material for: Immature HIV-1 assembles from Gag dimers leaving partial hexamers at lattice edges as potential substrates for proteolytic maturation
Source: Proc Natl Acad Sci U S A. 2021 Jan 4;118(3):e2020054118. doi: 10.1073/pnas.2020054118 (PMC7826355; doi:10.1073/pnas.2020054118)
Supplement: Supplementary File [file pnas.2020054118.sapp.pdf]

PNAS
www.pnas.org

Supplementary Information for
Immature HIV-1 assembles from Gag dimers leaving partial hexamers at lattice edges as potential substrates for proteolytic maturation

Aaron Tan[#], Alexander J. Pak[#], Dustin R. Morado, Gregory A. Voth*, & John A. G. Briggs*

# Equal contribution

* Corresponding authors: Gregory A. Voth / John A. G. Briggs
Email: gavoth@uchicago.edu / jbriggs@mrc-lmb.cam.ac.uk

This PDF file includes:

Figures S1 to S6
Table S1

www.pnas.org/cgi/doi/10.1073/pnas.2020054118

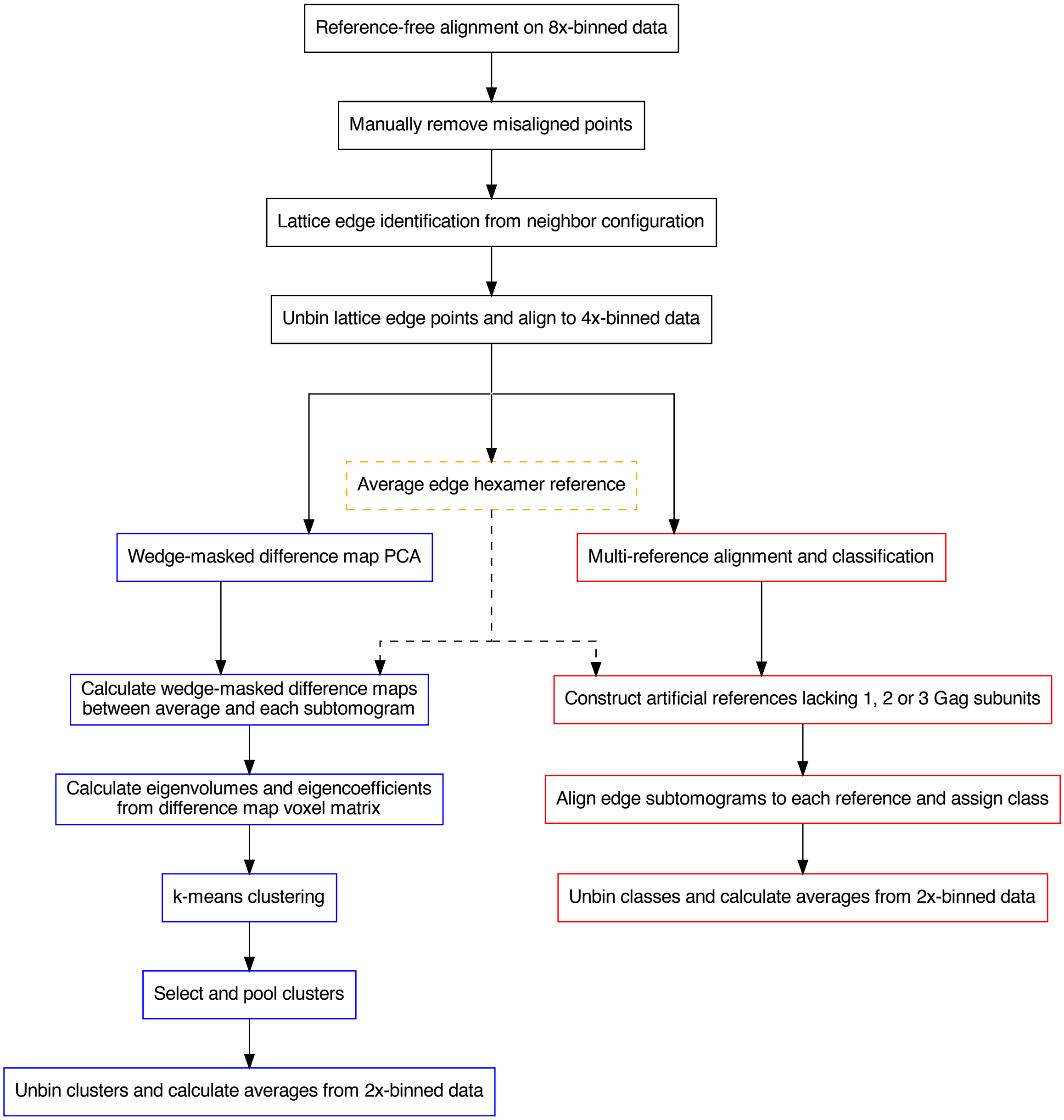

Fig. S1. Subtomogram alignment and classification workflow used to determine Gag lattice edge structures. A dataset of 8× binned binned subtomograms from (10), which had previously been aligned reference-free as described in Materials and Methods, was used as a starting point for manual removal of misaligned points (black boxes). An initial geometric identification and re-orientation of hexamers along lattice edges from the configuration of neighboring subtomograms was then performed, followed by extraction of subtomograms centered on the identified coordinates from 4× binned data (black boxes). An initial average reference containing all identified edge hexamers was constructed using 4× binned data as described in Materials and Methods (dashed yellow box). This reference was used to calculate wedge-masked difference maps against each subtomogram (blue boxes), and separately also to construct synthetic references for multireference alignment and classification (red boxes). These two classification approaches were carried out completely independently on the same input data.

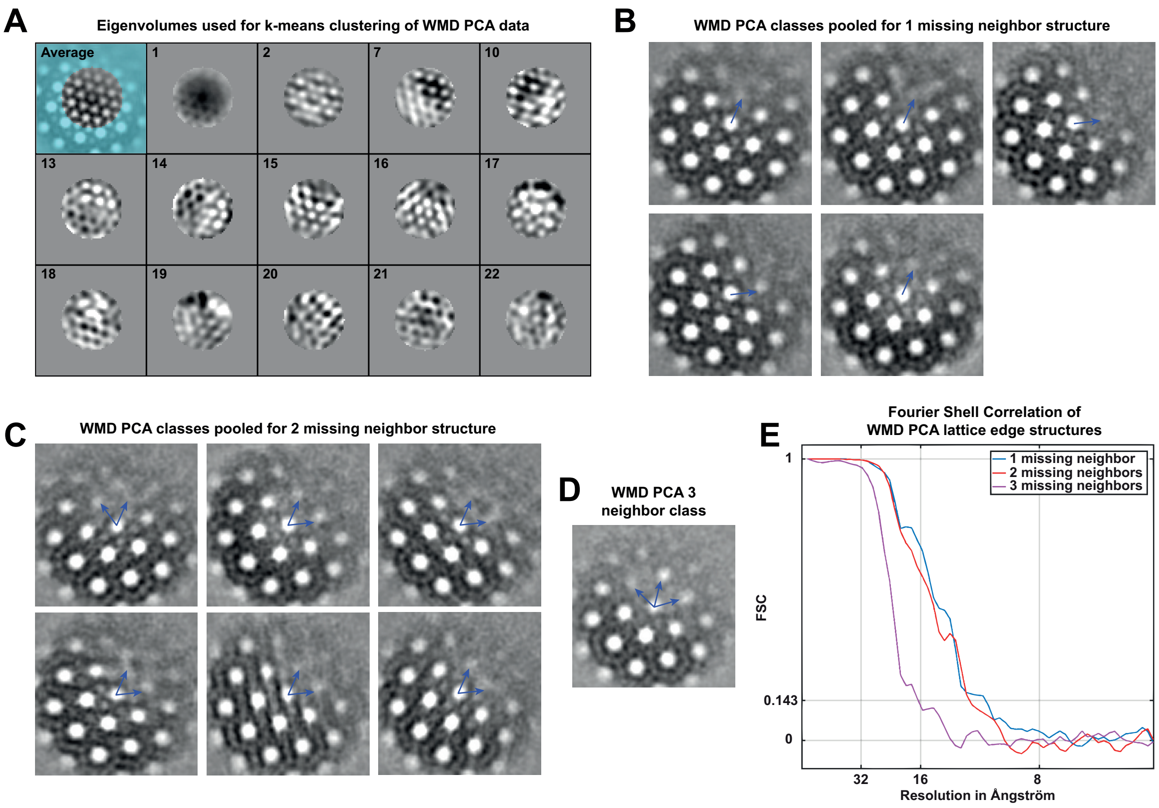

Fig. S2. Image processing details for WMD PCA classification of lattice edge hexamers. (A) Central XY slices through eigenvolumes selected as the principal components defining the lower-dimensional space onto which subtomograms were projected for classification, labelled with corresponding principal component number. The top left panel shows the average structure with an overlaid binary mask defining the voxels used for difference map calculation, with cyan regions not considered. (B) Classes from k-means clustering based on wedge-masked difference maps, corresponding to hexamers with 1 missing neighbor. Two classes were rotated by 60° relative to the other classes, corresponding to inaccuracies in the initial geometric orientation of the missing neighbor position (positions denoted by blue arrows extending from the central hexamer, see Materials and Methods). (C) As in B, for hexamers missing 2 neighbors. (D) As in B and C, for the single class of hexamers missing 3 neighbors. (E) Fourier shell correlation (FSC) curves between the odd and even half-datasets for each partial hexamer structure after further alignment with 2× binned data (see Materials and Methods).

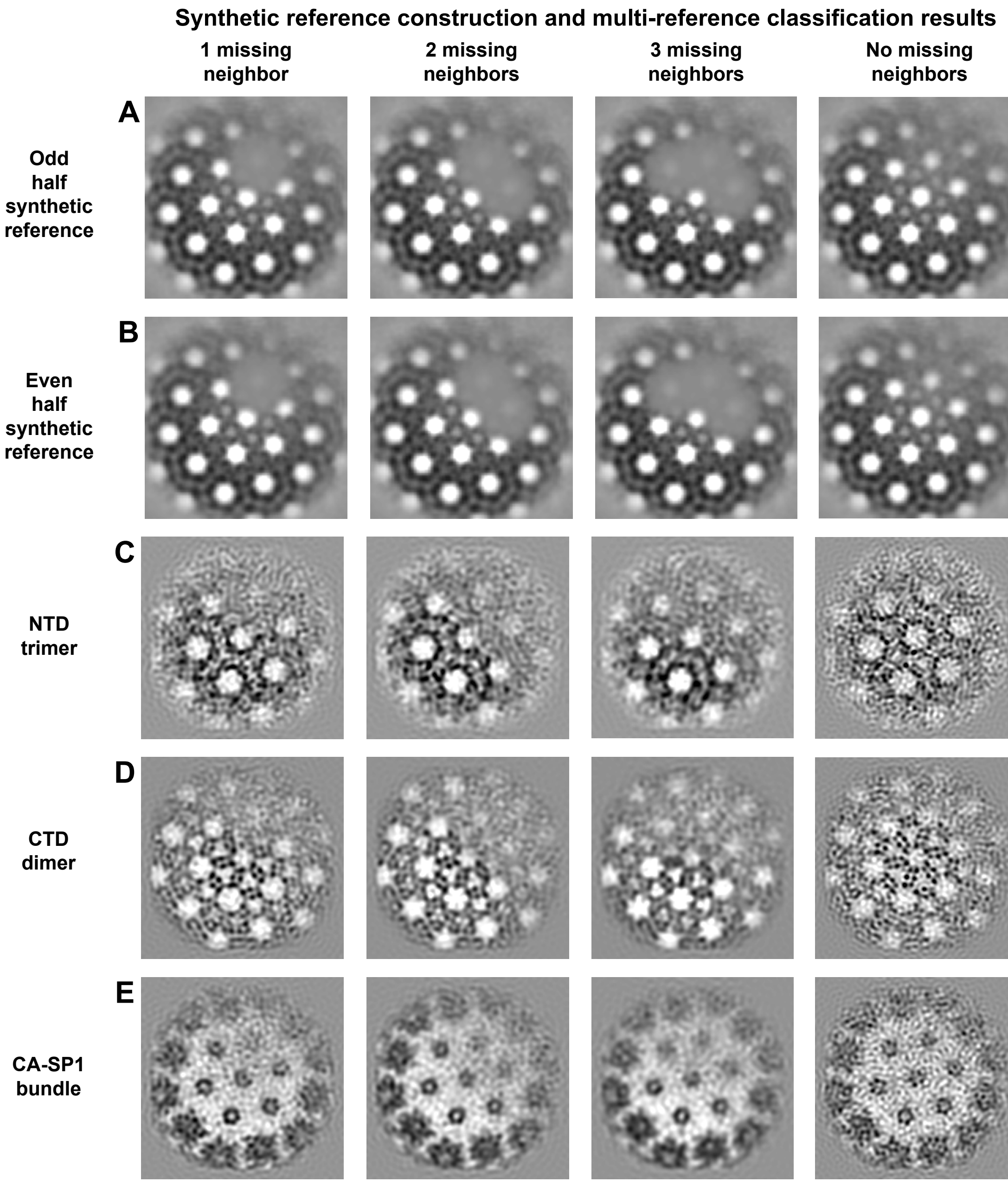

Fig. S3. Synthetic references and subtomogram alignment results from multi-reference subtomogram alignment and classification of Gag lattice edge hexamers missing different numbers of neighbors. Synthetic references (A-B) were constructed by down-weighting density corresponding to individual hexamer positions by masking as described in Materials and Methods. Panels (A) corresponds to the synthetic references constructed using the odd half-dataset average, and panel (B) correspond to those constructed using the even half-dataset average. (C) Orthoslices through the CA_{NTD}, (D) through the CA_{CTD} and (E) through the CA-SP1 helical bundle layers of the resulting final class averages from multi-reference alignment and classification are also shown for the classes corresponding to positions with 0, 1, 2 and 3 missing neighboring hexamers.

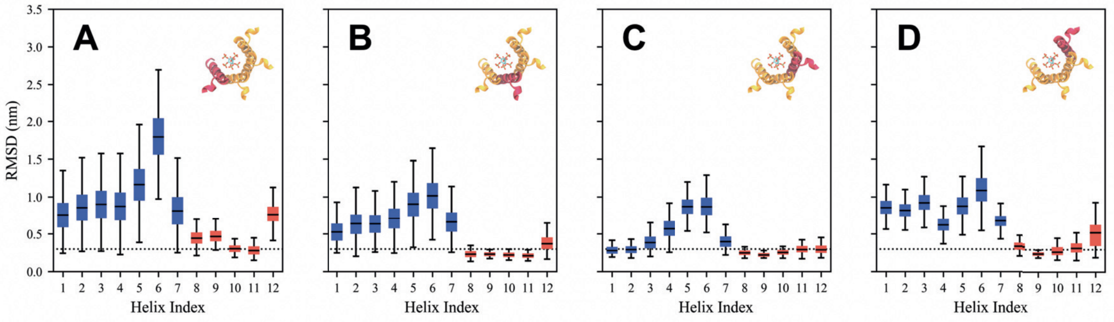

Fig. S4. Comparison of the root mean squared deviation (RMSD) with respect to an atomic model (PDB 5L93) of a CA-SP1 monomer within an incomplete hexamer missing 2 Gag subunits and the clockwise-most monomer missing CA_{NTD} trimer contacts and maintaining CA_{CTD} dimer contacts (same as Fig. 3C). From (A-D), different monomers throughout the incomplete hexamer are analyzed with the representative CA_{CTD}/SP1 helix colored in red in the inset. Each box bounds the upper and lower quartiles with the central line indicating the median, while the whiskers show the extrema of the distributions. Blue (red) boxes refer to the analyzed CA_{NTD} (CA_{CTD}) monomer. The dotted line marks a RMSD of 0.3 nm and serves as a guide to the eye (see also Fig. 3). Note that the RMSD of helix 12, the CA_{CTD}/SP1 helix, is larger for edge monomers compared to internal monomers, suggesting that the edge helices may have increased probability for uncoiling.

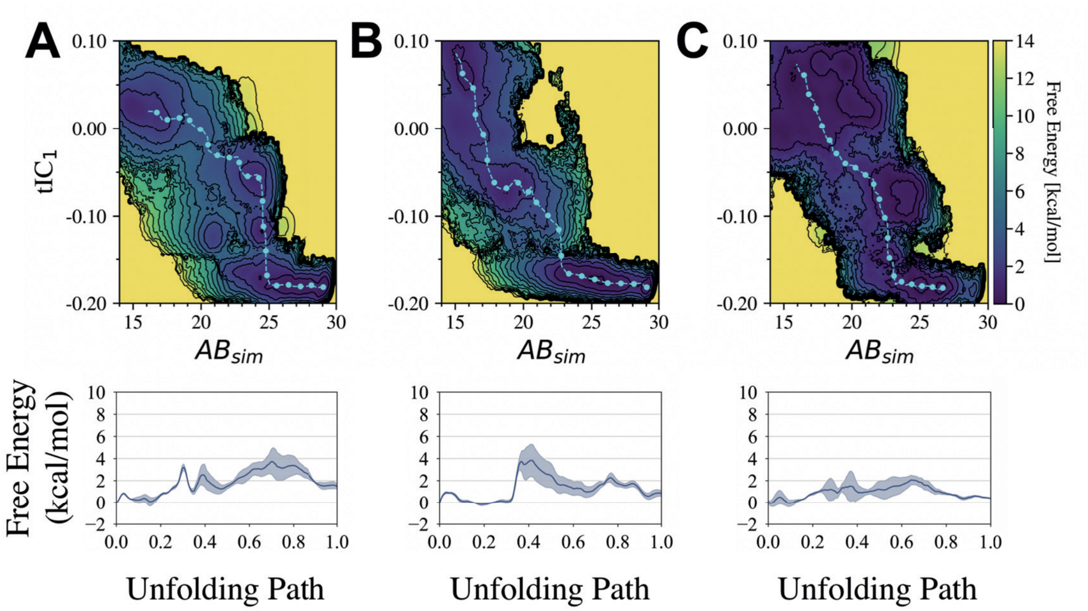

Fig. S5. Comparison of free energy surfaces projected onto the alpha-beta similarity (AB_{sim}) and first time-structure independent component (tIC_1) for 6HBs in the absence of IP_6. We compare (A) a helix in a complete hexamer to (B, C) helices in an incomplete hexamer missing 2 neighboring CA-SP1 monomers, where (B) is a helix between two neighboring helices and (C) is the outer helix (with V362 and A366 exposed to solvent). Each respective minimum free energy path is depicted as a cyan line with cyan dots and quantified in each of the bottom plots.

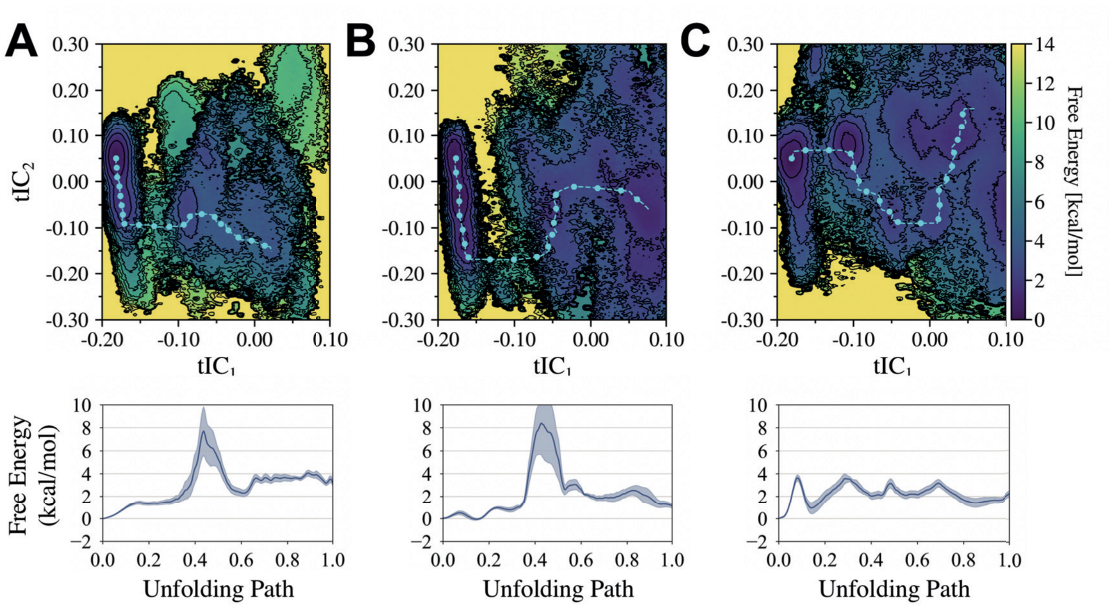

Fig. S6. An alternate comparison of free energy surfaces projected onto the first (tIC_1) and second (tIC_2) time-structure independent components. We compare (A) a helix in a complete hexamer to (B, C) helices in an incomplete hexamer missing 2 neighboring CA-SP1 monomers, where (B) is a helix between two neighboring helices and (C) is the outer helix (with V362 and A366 exposed to solvent). Each respective minimum free energy path is depicted as a cyan line with cyan dots and quantified in each of the bottom plots. In each case, the 6HB is coordinated by IP_6.

Table S1 Image processing statistics for all lattice edge classes.

Class		1 missing neighbor	2 missing neighbors	3 missing neighbors	No missing neighbors
**WMD PCA classification**	**Viruses**	483	479	475	-
	**Asymmetric units Set A**	5,009	5,935	1,645	-
	**Asymmetric units Set B**	5,008	5,935	1,645	-
	**Final resolution (0.143 FSC) in Å**	11.8	11.2	16.2	-
	**Map pixel size in Å**	2.7	2.7	2.7	-
**Multi-reference alignment and classification**	**Viruses**	484	484	484	484
	**Asymmetric units Set A**	6,822	6,671	6,136	8,938
	**Asymmetric units Set B**	6,821	6,671	6,136	8,939
	**Final resolution (0.143 FSC) in Å**	10.2	10.9	11.7	9.2
	**Map pixel size in Å**	2.7	2.7	2.7	2.7
